# Using Web-Based Questionnaires and Obstetric Records to Assess General Health Characteristics Among Pregnant Women: A Validation Study

**DOI:** 10.2196/jmir.3847

**Published:** 2015-06-16

**Authors:** Marleen MHJ van Gelder, Naomi PE Schouten, Peter JFM Merkus, Chris M Verhaak, Nel Roeleveld, Jolt Roukema

**Affiliations:** ^1^ Department for Health Evidence Radboud Institute for Health Sciences Radboud university medical center Nijmegen Netherlands; ^2^ Radboud REshape Innovation Center Radboud university medical center Nijmegen Netherlands; ^3^ Division of Respiratory Medicine Department of Pediatrics, Radboudumc Amalia Children’s Hospital Radboud university medical center Nijmegen Netherlands; ^4^ Department of Pediatrics Canisius Wilhelmina Hospital Nijmegen Netherlands; ^5^ Department of Medical Psychology Radboud university medical center Nijmegen Netherlands; ^6^ Department of Pediatrics Radboudumc Amalia Children’s Hospital Radboud university medical center Nijmegen Netherlands

**Keywords:** questionnaires, medical records, validation studies, pregnancy, chronic disease, allergens, blood pressure, Internet, PRIDE Study

## Abstract

**Background:**

Self-reported medical history information is included in many studies. However, data on the validity of Web-based questionnaires assessing medical history are scarce. If proven to be valid, Web-based questionnaires may provide researchers with an efficient means to collect data on this parameter in large populations.

**Objective:**

The aim of this study was to assess the validity of a Web-based questionnaire on chronic medical conditions, allergies, and blood pressure readings against obstetric records and data from general practitioners.

**Methods:**

Self-reported questionnaire data were compared with obstetric records for 519 pregnant women participating in the Dutch PRegnancy and Infant DEvelopment (PRIDE) Study from July 2011 through November 2012. These women completed Web-based questionnaires around their first prenatal care visit and in gestational weeks 17 and 34. We calculated kappa statistics (κ) and the observed proportions of positive and negative agreement between the baseline questionnaire and obstetric records for chronic conditions and allergies. In case of inconsistencies between these 2 data sources, medical records from the woman’s general practitioner were consulted as the reference standard. For systolic and diastolic blood pressure, intraclass correlation coefficients (ICCs) were calculated for multiple data points.

**Results:**

Agreement between the baseline questionnaire and the obstetric record was substantial (κ=.61) for any chronic condition and moderate for any allergy (κ=.51). For specific conditions, we found high observed proportions of negative agreement (range 0.88-1.00) and on average moderate observed proportions of positive agreement with a wide range (range 0.19-0.90). Using the reference standard, the sensitivity of the Web-based questionnaire for chronic conditions and allergies was comparable to or even better than the sensitivity of the obstetric records, in particular for migraine (0.90 vs 0.40, *P*=.02), asthma (0.86 vs 0.61, *P*=.04), inhalation allergies (0.92 vs 0.74, *P*=.003), hay fever (0.90 vs 0.64, *P*=.001), and allergies to animals (0.89 vs 0.53, *P*=.01). However, some overreporting of allergies was observed in the questionnaire and for some nonsomatic conditions sensitivity of both measurement instruments was low. The ICCs for blood pressure readings ranged between 0.72 and 0.92 with very small mean differences between the 2 methods of data collection.

**Conclusions:**

Web-based questionnaires can be used to validly collect data on many chronic disorders, allergies, and blood pressure readings among pregnant women.

## Introduction

Self-reported methods of data collection are often applied in large-scale medical or biomedical studies for efficiency reasons. In these studies, it may not be feasible to conduct clinical measurements on all participants. Therefore, paper-and-pencil questionnaires or telephone interviews were traditionally used to gather information on the study variables. Nowadays, these modes of data collection are increasingly being substituted by Web-based questionnaires. However, knowledge on the validity of data collected with Web-based questionnaires is limited [1], although the quality of the data on a number of traditional epidemiologic risk factors, including body weight [2-4], smoking [5], alcohol consumption [6], and energy and macronutrient intake [7,8], is reported to be high. Medical history is included as an exposure or potential confounding factor in many studies and Web-based questionnaires may be an efficient way to collect these data in large samples of participants, if proven to be valid.

Most validation studies on medical history collected through self-reported methods has focused on chronic conditions, in particular cardiovascular diseases [9-15], diabetes [10,12-16], cancer [11,17,18], and asthma [10,13,14,19,20]. Agreement between self-reports and medical records differed among these studies and was affected by study methodology, target population, condition of interest, and the statistical analyses. In general, agreement was good for conditions that have clear diagnostic criteria, but it was low to moderate for conditions that are less serious or more complex to diagnose. Accordingly, discordance between questionnaires and biochemical measures or patch testing for allergic conditions or atopy is substantial [21-23]. Data on the validity of self-report on the results of common measurements taken during health care visits, such as blood pressure readings and hemoglobin levels, are very limited.

To the best of our knowledge, only Landkroon et al [24] compared data on medical history from a Web-based questionnaire with a “reference standard,” but this study was too small (N=106) to produce robust estimates for levels of agreement. Therefore, the aim of this study was to assess the validity of a Web-based questionnaire on chronic conditions, allergies, and blood pressure readings among pregnant women by comparing the questionnaire data to obstetric records and data from general practitioners (GPs).

## Methods

### Setting

The Dutch prenatal care system is unique in the Western world. In the Netherlands, midwives are qualified to provide full prenatal care to all women with uncomplicated pregnancies and deliveries. The first prenatal care visit, which may be scheduled without referral of a general practitioner, usually takes place in gestational weeks 8 to 10 and frequent contacts are scheduled throughout pregnancy. Women are referred to a secondary or tertiary midwife or gynecologist in case of risk factors or complications. In 2013, 85% of pregnant women started their prenatal care in a primary care setting [25].

### Study Population

We used data from the PRegnancy and Infant DEvelopment (PRIDE) Study, an ongoing, prospective cohort study that enrolls Dutch women early in pregnancy. The PRIDE Study started enrollment in July 2011 in the Nijmegen region and aims at including more than 150,000 pregnancies to study a broad range of research questions pertaining to maternal and child health. Details on the study design are described elsewhere [26]. Briefly, pregnant women aged 18 years and older were invited to participate in the PRIDE Study by their midwife or gynecologist just before or during their first prenatal care visit. They were asked to complete Web-based questionnaires at baseline, in gestational weeks 17 (questionnaire 2) and 34 (questionnaire 3), as well as 2 and 6 months after the estimated date of delivery. The baseline questionnaire was completed between weeks 6 and 16 of gestation. Researchers from various medical disciplines selected, modified, and tailored existing, validated paper-based questionnaires or parts thereof to fit our Web-based application. Paper-based questionnaires were available for women who could not or did not want to participate through the Internet (n=1; excluded from this study). Questions were asked on demographic factors, reproductive history, maternal health, lifestyle factors, and occupational exposures. Furthermore, consent was asked for review of medical records to enrich the PRIDE Study database with detailed clinical information.

### Data Collection

Through the baseline questionnaire, data on medical history were collected. Women were asked gateway questions to assess chronic conditions (“Do you have a chronic or long-term illness that was diagnosed by a medical doctor” followed by some examples of chronic conditions) and allergies (“Do you have an allergy or eczema?”). These questions were followed by multiple-choice questions with blank options to specify the chronic condition or allergy among those who answered positively to the relevant gateway question. Chronic conditions reported in other parts of the baseline questionnaire (eg, as causes for subfertility or as indications for medication use) were included in the analysis as well. In each prenatal questionnaire, we asked for the date of the most recent prenatal care visit, whether blood pressure was measured during this visit, and if so, for the systolic and diastolic blood pressure readings in mm Hg. A screenshot of the relevant parts of the questionnaires is provided in Multimedia Appendix 1.

A pretested, standardized case report form (CRF) was used to abstract data from the obstetric records of women who gave consent for medical record review. For logistical reasons, obstetric records were only reviewed in participating study centers in the Nijmegen region (7 midwifery practices and 1 academic hospital). Using the CRF, 2 medically trained abstracters collected data from the obstetric records on medical history, including chronic conditions, allergies, and pregnancy history, the pregnancy itself, anthropometrical measures including blood pressure taken during pregnancy, and pregnancy outcome, not all of which were included in this validation study.

Preexisting medical conditions are self-reported by the pregnant woman during the first prenatal care visit and are usually only recorded in the obstetric record by the prenatal care provider if deemed important for the course of pregnancy or the delivery [27]. As a consequence, obstetric records may not be a suitable reference standard for self-reported chronic conditions and allergies. Therefore, information on the diagnosis of chronic conditions and allergies was obtained from the woman’s GP in case of inconsistencies between the questionnaire and the obstetric record for reasons of efficiency.

Chronic conditions were classified and coded according to the World Health Organization’s *International Classification of Diseases, Tenth Revision* [28]. Allergies were ordered into 6 mutually exclusive categories: (1) inhalation allergies (hay fever, allergies to animals, and house dust mite allergy), (2) food allergies, (3) allergic contact dermatitis (allergies to metal, fragrance hypersensitivity, plaster allergy, and latex allergy), (4) insect sting allergy, (5) medication allergies, and (6) other allergies.

### Statistical Analysis

**Figure 1:**
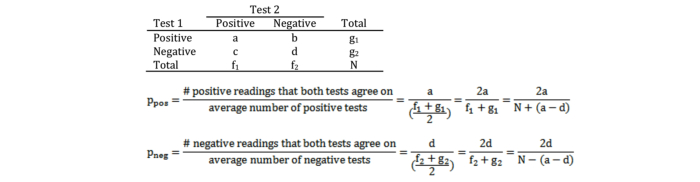
Calculation of positive and negative agreement between two tests.

Only PRIDE Study participants with complete information on chronic conditions, allergies, and blood pressure during the most recent prenatal care visit in the baseline questionnaire who gave consent to review their medical records were included in this validation study. For chronic conditions and allergies with at least 5 cases in either the questionnaire or the obstetrical record, we calculated kappa statistics (κ) to quantify agreement between the baseline questionnaire and the obstetric record regarding chronic conditions and allergies. We also calculated the observed proportions of positive and negative agreement (p_pos_ and p_neg_, respectively) because kappa is strongly affected by imbalances in marginal totals (ie, a low kappa despite a high level of agreement) [29]. The calculation of p_pos_ and p_neg_ is shown in Figure 1 [30].

To determine which method of data collection was most valid to collect information on chronic conditions and allergies among pregnant women, sensitivity and specificity were calculated with GP data until the date of completion of the baseline questionnaire as our reference standard. When GP data were unavailable, pharmacy records were screened for diagnoses of chronic conditions or allergies and for medication dispensed that was indicative for chronic conditions or allergies. In addition to the discordant questionnaire–obstetric record pairs, women with positive scores on both the Web-based questionnaire and the obstetric record were included in these calculations as true positives. Likewise, women with negative scores on both methods were included as true negatives. We assessed potential differences in sensitivity and specificity between the questionnaires and the obstetric records using chi-square tests.

For the validity analyses regarding blood pressure readings, only women with an exact match between the date of the most recent prenatal care visit reported in any of the prenatal questionnaires and a visit date recorded in the obstetric record were included to be certain that both data sources referred to the same measurement. Intraclass correlation coefficients (ICCs) with 95% confidence intervals (CIs) for systolic blood pressure (SBP) and diastolic blood pressure (DBP) were calculated using 2-way mixed effects models (single measure). To assess absolute agreement and potential differences in bias within the SBP and DBP range, we plotted the difference in blood pressure readings between the questionnaire and the obstetric record (y-axis) against the mean of the 2 methods of data collection (x-axis) according to the Bland-Altman technique [31]. In secondary analyses, we included all women who reported the most recent prenatal care visit date in the questionnaire within 5 days of a visit date recorded in the obstetric record. All statistical analyses were performed using IBM SPSS version 20 (IBM Corp, Armonk, NY, USA), except for p_pos_ and p_neg_, which were calculated in Microsoft Office Excel 2007 (Microsoft Corp, Redmond, WA, USA).

## Results

**Figure 2:**
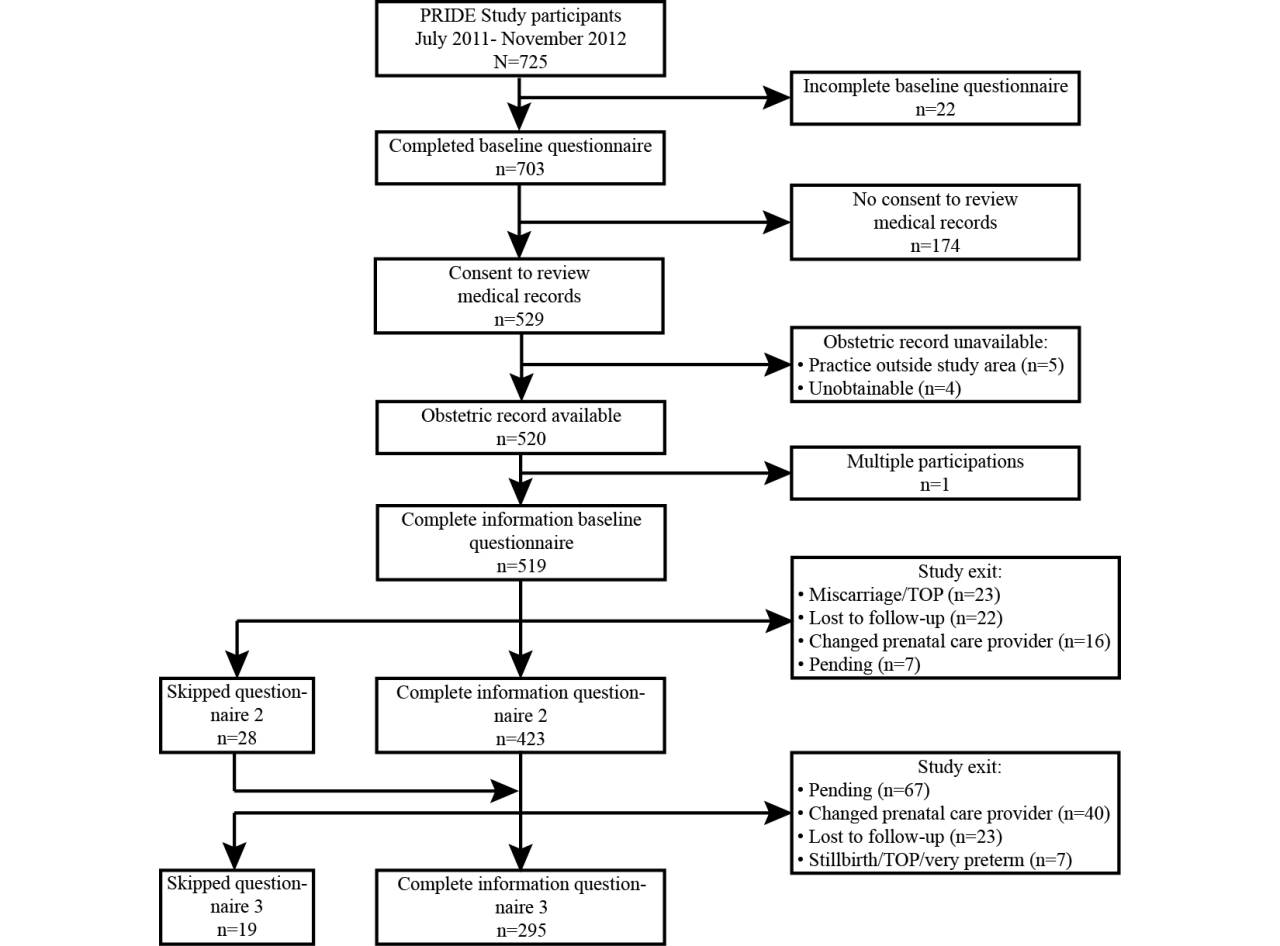
Flow chart of study participants.

Women enrolled in the PRIDE Study between July 2011 through November 2012 were eligible for this study (N=725). The overall participation rate in the PRIDE Study was 42.90% (725/1690) during this time period. Figure 2 shows the flow of participants. Of the 725 women enrolled during the study period, 22 (3.0%) only completed a few sections of the baseline questionnaire, mostly because of technical issues in the first weeks of enrollment. Among those with complete baseline questionnaires, 24.8% (174/703) did not give consent for medical record review. Furthermore, 10 women were excluded because their obstetric records were not available (n=9) or they participated with multiple pregnancies in the PRIDE Study (n=1). Therefore, 519 women were included in this validation study. Compared with the women who did not give consent to obtain medical records, women participating in this validation study were more likely to have a lower level of education (*P*=.03) and to be obese (*P*=.06; Table 1). Furthermore, women who did not give consent for medical record review were more likely to have completed the baseline questionnaire before their first prenatal care visit compared to women included in the validation study (*P*=.02). We did not observe substantial differences in maternal age, country of birth, gravidity, and gestational age at inclusion between these 2 groups. Regarding the blood pressure readings, follow-up information was not available for all participants for several reasons: (1) they did not reach the gestational week for administration of questionnaire 2 or 3 yet at the date of obstetric record review; (2) they had a miscarriage, stillbirth, termination of pregnancy (TOP), or very preterm birth; or (3) they skipped questionnaire 2 or 3, were lost to follow-up, or changed prenatal care provider resulting in incomplete obstetric records.

**Table 1:** Characteristics of PRIDE Study participants included in this validation study and participants who did not give consent for review of medical records.

Characteristic		Participants in validation study, n (%) (N=519)^a^	No consent for medical record review, n (%) (N=174)^a^	*P* ^b^
**Maternal age at inclusion (years)**				.45
	<25	15 (2.9)	2 (1.1)	
	25-29	172 (33.1)	52 (29.9)	
	30-34	238 (45.9)	88 (50.6)	
	≥35	94 (18.1)	32 (18.4)	
**Maternal country of birth**				.20
	Netherlands	483 (93.1)	154 (88.5)	
	Other	31 (6.0)	15 (8.6)	
**Maternal level of education** ^c^				.03
	Low/intermediate	107 (20.6)	22 (12.6)	
	High	407 (78.4)	147 (84.5)	
**Gravidity**				.62
	0	232 (44.7)	74 (42.5)	
	≥1	287 (55.3)	100 (57.5)	
**BMI before pregnancy** ^d^				.06
	Underweight (<18.5 kg/m^2^)	15 (2.9)	2 (1.1)	
	Normal (18.5-24.9 kg/m^2^)	336 (64.7)	122 (70.1)	
	Overweight (25.0-29.9 kg/m^2^)	90 (17.3)	27 (15.5)	
	Obese (≥30 kg/m^2^)	33 (6.4)	3 (1.7)	
	Height/weight unknown	45 (8.7)	20 (11.5)	
**Gestational age at inclusion (weeks)**				.17
	<8	96 (18.5)	41 (23.6)	
	8-10	178 (34.3)	48 (27.6)	
	>10	245 (47.2)	85 (48.9)	
**Timing of baseline questionnaire**				.02
	Completed before first prenatal care visit	131 (25.2)	60 (34.5)	
	Completed after first prenatal care visit	388 (74.8)	114 (65.5)	

^a^ Numbers may not add up to total group size due to missing values.

^b^ Difference between the 2 groups using chi-square tests.

^c^ High level of education: completed higher vocational education or university.

^d^ Body mass index (BMI) derived from self-reported height and weight.

Of the 519 participants, 118 (22.7%) women reported having a chronic condition in the baseline questionnaire, whereas chronic conditions were recorded in the obstetric records of 105 (20.2%) women. Overall, agreement between the Web-based questionnaire and the obstetric record was substantial for any chronic condition (κ=.61; Table 2) with a higher p_neg_ (0.92) than p_pos_ (0.69). Level of agreement differed between the specific chronic conditions with relatively high levels of agreement for endocrine, nutritional, and metabolic diseases (κ=.72) and in particular for thyroid disease (κ=.90), epilepsy (κ=.89), and diseases of the genitourinary tract (κ=.72). However, for a number of conditions, including migraine (κ=.30), diseases of the circulatory system (κ=.25), and irritable bowel syndrome (κ=.39), agreement between the questionnaire and the obstetric record was poor. For all specific conditions, the p_neg_ was high (range 0.98-1.00), but the p_pos_ followed a pattern comparable to the kappa statistic.

**Table 2:** Agreement between data from the Web-based questionnaire and obstetric record for chronic conditions (n=519).

Chronic condition	Questionnaire positive, n		Questionnaire negative, n		κ	p_pos_^a^	p_neg_^b^
	Record positive	Record negative	Record positive	Record negative			
Any chronic condition	77	41	28	373	.61	0.69	0.92
Diseases of the blood and blood-forming organs	2	0	0	517	—	—	—
Thalassemia	1	0	0	518	—	—	—
Immunodeficiency	1	0	0	518	—	—	—
Endocrine, nutritional, and metabolic diseases	15	5	6	493	.72	0.73	0.99
Thyroid disease	9	1	1	508	.90	0.90	1.00
Polycystic ovarian syndrome	5	3	5	506	.55	0.56	0.99
Hypercholesterolemia	0	1	0	518	—	—	—
Periodic fever syndrome	1	0	0	518	—	—	—
Mental and behavioral disorders	7	6	4	502	.57	0.58	0.99
Depression/anxiety	5	5	4	505	.52	0.53	0.99
Posttraumatic stress disorder	1	1	0	517	—	—	—
ADD/ADHD^c^	1	1	0	517	—	—	—
Diseases of the nervous system	12	17	4	486	.51	0.53	0.98
Multiple sclerosis	1	0	0	518	—	—	—
Epilepsy	4	0	1	514	.89	0.89	1.00
Migraine	4	17	1	497	.30	0.31	0.98
Tension-type headache	1	0	1	517	—	—	—
Chronic fatigue syndrome	2	0	1	516	—	—	—
Diseases of the circulatory system	1	1	5	512	.25	0.25	0.99
Hypertension	0	0	3	516	—	—	—
Cardiac arrhythmia	0	1	1	517	—	—	—
Raynaud syndrome	1	0	1	517	—	—	—
Diseases of the respiratory system	19	17	5	478	.61	0.63	0.98
Asthma	19	17	5	478	.61	0.63	0.98
Diseases of the digestive system	8	4	6	501	.61	0.62	0.99
Crohn disease	2	0	1	516	—	—	—
Ulcerative colitis	3	0	0	516	—	—	—
Irritable bowel syndrome	3	4	5	507	.39	0.40	0.99
Diseases of the skin and subcutaneous tissue	4	7	1	507	.49	0.50	0.99
Psoriasis	4	5	0	510	.61	0.62	1.00
Rosacea	0	2	1	516	—	—	—
Diseases of the musculoskeletal system and connective tissue	9	4	4	502	.68	0.69	0.99
Rheumatoid arthritis	2	2	0	515	—	—	—
Sjögren syndrome	1	0	0	518	—	—	—
Ankylosing spondylitis	1	0	1	517	—	—	—
Hypermobility	1	1	3	514	—	—	—
Fibromyalgia	2	2	1	514	—	—	—
Complex regional pain syndrome	1	0	0	518	—	—	—
Diseases of the genitourinary tract	4	1	2	512	.72	0.73	1.00
Endometriosis	1	1	1	516	—	—	—
Lichen sclerosis	3	0	1	515	—	—	—

^a^ Observed proportion of positive agreement.

^b^ Observed proportion of negative agreement.

^c^ ADD: attention deficit disorder; ADHD: attention deficit hyperactivity disorder.

Allergies were reported by 229 of 519 (44.1%) women in the baseline questionnaire and recorded in the obstetric record of 168 (32.4%) women. In Table 3, agreement between the Web-based questionnaire and the obstetric record is shown for the mutually exclusive groups of allergies and selected specific allergies. For any allergy, agreement between the questionnaire and the obstetric record was moderate (κ=.51) with a p_pos_ and p_neg_ of 0.70 and 0.81, respectively. The kappa values for the groups of allergies ranged between 0.21 (insect sting allergy) and 0.66 (drug allergies) and between 0.33 (fragrance hypersensitivity) and 0.73 (latex allergy) for the specific types of allergies. House dust mite allergy, latex allergy, and drug allergies were more often reported in the obstetric record than in the questionnaire. Again, the p_neg_ (range 0.81-1.00) was higher than the p_pos_ (range 0.19-0.73) for all groups of allergies or specific allergies included.

**Table 3:** Agreement between data from a Web-based questionnaire and obstetric record for allergies (n=519).

Allergy	Questionnaire positive, n		Questionnaire negative, n		κ	p_pos_^a^	p_neg_^b^
	Record positive	Record negative	Record positive	Record negative			
Any allergy	138	91	30	260	.51	0.70	0.81
Inhalation allergies	80	83	10	346	.53	0.63	0.88
Hay fever	53	53	3	410	.60	0.65	0.94
Allergies to animals	21	45	7	446	.40	0.45	0.94
House dust mite allergy	15	15	22	467	.41	0.45	0.96
Food allergies	15	39	1	464	.40	0.43	0.96
Allergic contact dermatitis	33	73	11	402	.36	0.44	0.91
Allergies to metal	11	37	3	468	.33	0.35	0.96
Fragrance hypersensitivity	6	51	0	462	.17	0.19	0.95
Plaster allergy	9	16	7	487	.42	0.44	0.98
Latex allergy	4	0	3	512	.73	0.73	1.00
Insect sting allergy	3	20	1	495	.21	0.22	0.98
Drug allergies	26	9	15	469	.66	0.68	0.98
Other allergies	2	3	0	514	.57	0.57	1.00

^a^ Observed proportion of positive agreement.

^b^ Observed proportion of negative agreement.

Regarding the 254 women with an inconsistency between the Web-based questionnaire and the obstetric record for chronic conditions or allergies, complete GP data were obtained for 194 (76.4%) women; the GP was unknown for 12 women, 21 women were not registered with the GP whose name was provided, the GP did not respond to our multiple data requests for 25 women, and GP records were incomplete for 2 women. For 7 women lacking GP data, the diagnosis of a chronic disorder was ascertained from their pharmacy records. Generally, sensitivity was better for the Web-based questionnaire than for the obstetric record when compared to GP data (Table 4), specifically for migraine (0.90 vs 0.40, *P*=.02), asthma (0.86 vs 0.61, *P*=.04), any allergy (0.96 vs 0.85, *P*=.007), inhalation allergies (0.92 vs 0.74, *P*=.003), hay fever (0.90 vs 0.64, *P*=.001), and allergies to animals (0.89 vs 0.53, *P*=.01). For a number of chronic conditions, including mental and behavioral disorders, depression/anxiety, and irritable bowel syndrome, sensitivity of both measurement instruments was low. Overall, specificity of the Web-based questionnaire and the obstetric record was high. However, specificity of the questionnaire was slightly lower than specificity of the obstetric record for a number of (groups of) allergies, including any allergy (0.74 vs 0.85, *P*=.009), inhalation allergies (0.83 vs 0.93, *P*<.001), hay fever (0.91 vs 0.96, *P*=.001), allergies to animals (0.91 vs 0.97, *P*<.001), food allergies (0.92 vs 0.98, *P*<.001), allergic contact dermatitis (0.84 vs 0.94, *P*<.001), allergies to metal (0.94 vs 0.99, *P*<.001), and fragrance hypersensitivity (0.91 vs 0.99, *P*<.001).

**Table 4:** Validity comparisons of chronic conditions and allergies among pregnant women: Web-based questionnaires and obstetric records compared to GP records.

Condition or allergy	n	Sensitivity			Specificity		
		Questionnaire	Record	*P* ^a^	Questionnaire	Record	*P* ^a^
Any chronic condition	496	0.83	0.74	.14	0.93	0.92	.79
Endocrine, nutritional, and metabolic diseases	515	0.76	0.70	.73	1.00	0.99	.41
Thyroid disease	518	0.77	0.69	.66	1.00	1.00	>.99
Polycystic ovarian syndrome	516	0.67	0.83	.52	1.00	0.99	.41
Mental and behavioral disorders	516	0.45	0.27	.39	0.99	0.99	.78
Depression/anxiety	516	0.50	0.30	.37	0.99	0.99	.48
Diseases of the nervous system	513	0.85	0.65	.15	0.99	1.00	.09
Epilepsy	519	0.80	1.00	.32	1.00	1.00	>.99
Migraine	514	0.90	0.40	.02	0.99	1.00	.03
Diseases of the respiratory system	511	0.86	0.61	.04	0.99	0.99	.76
Asthma	511	0.86	0.61	.04	0.99	0.99	.76
Diseases of the digestive system	517	0.58	0.67	.68	0.99	0.99	.74
Irritable bowel syndrome	517	0.33	0.33	>.99	0.99	0.99	.74
Diseases of the skin and subcutaneous tissue	519	0.69	0.38	.12	1.00	1.00	.16
Psoriasis	519	0.80	0.40	.08	1.00	1.00	.32
Diseases of the musculoskeletal system and connective tissue	515	1.00	0.82	.15	1.00	1.00	.16
Diseases of the genitourinary tract	518	0.80	1.00	.32	1.00	1.00	.32
Any allergy	494	0.96	0.85	.007	0.74	0.85	.009
Inhalation allergies	496	0.92	0.74	.003	0.83	0.93	<.001
Hay fever	508	0.90	0.64	.001	0.91	0.96	.001
Allergies to animals	509	0.89	0.53	.01	0.91	0.97	<.001
House dust mite allergy	512	0.58	0.65	.58	0.98	0.97	.44
Food allergies	511	1.00	0.86	.32	0.92	0.98	<.001
Allergic contact dermatitis	502	1.00	1.00	>.99	0.84	0.94	<.001
Allergies to metal	508	1.00	1.00	>.99	0.94	0.99	<.001
Fragrance hypersensitivity	507	1.00	1.00	>.99	0.91	0.99	<.001
Plaster allergy	511	1.00	1.00	>.99	0.96	0.97	.36
Drug allergies	515	0.84	0.95	.30	0.97	0.96	.32

^a^ Difference between the 2 modes of data collection using chi-square tests.

For 4 chronic conditions, additional self-reports were identified in the questions about causes of subfertility that preceded the chronic condition question (polycystic ovarian syndrome [n=7] and endometriosis [n=2]) and through medication use (rosacea [n=2] and lichen sclerosis [n=1]). When these women were considered as not having reported these chronic conditions, agreement between the Web-based questionnaire and the obstetric record decreased, except for skin diseases. Furthermore, it decreased the sensitivity of the questionnaire, especially for endocrine diseases (0.67), polycystic ovarian syndrome (no true positive subjects), and diseases of the genitourinary tract (0.67).

Analyses on the validity of the Web-based questionnaires for blood pressure readings could not be conducted on the complete study sample. At baseline, 123 of 519 (23.7%) women did not have a prenatal care visit yet and, therefore, no valid blood pressure measurement (Table 5). Among women with a prenatal care visit, no match on visit date was established for 91 of 396 (23.0%), 65 of 423 (15.4%), and 32 of 295 (10.8%) women for the baseline questionnaire, questionnaire 2, and questionnaire 3, respectively. Furthermore, a substantial proportion of women whose blood pressure was measured could not remember the blood pressure readings (baseline questionnaire: 27.9%, 76/272; questionnaire 2: 28.4%, 93/328; questionnaire 3: 19.1%, 50/262). Of the women included at baseline and eligible for the reliability analyses of the follow-up questionnaires, 78.6% (121/154) and 84.6% (88/104) were included for questionnaires 2 and 3, respectively. Out of the 142 women included for questionnaire 2 and eligible for the analysis of questionnaire 3, 128 (90.1%) were included for questionnaire 3.

**Table 5:** Validity analyses comparing Web-based questionnaires and obstetric records for systolic and diastolic blood pressure readings: sample description and intraclass correlation coefficients.

Characteristic		Baseline questionnaire (n=519)	Questionnaire 2 (n=423)	Questionnaire 3 (n=295)
Did not have prenatal care visit yet, n		123	0	0
No match date prenatal care visit, n		91	65	32
Questionnaire: blood pressure not measured, n		33	30	1
Questionnaire: blood pressure unknown, n		76	93	50
Obstetric record: blood pressure not recorded, n		11	12	1
Included in validity analyses, n		185	223	211
**Blood pressure, ICC (95% CI)**				
	Systolic	0.72 (0.65-0.79)	0.92 (0.89-0.94)	0.90 (0.88-0.93)
	Diastolic	0.79 (0.73-0.84)	0.91 (0.88-0.93)	0.89 (0.86-0.91)

**Figure 3:**
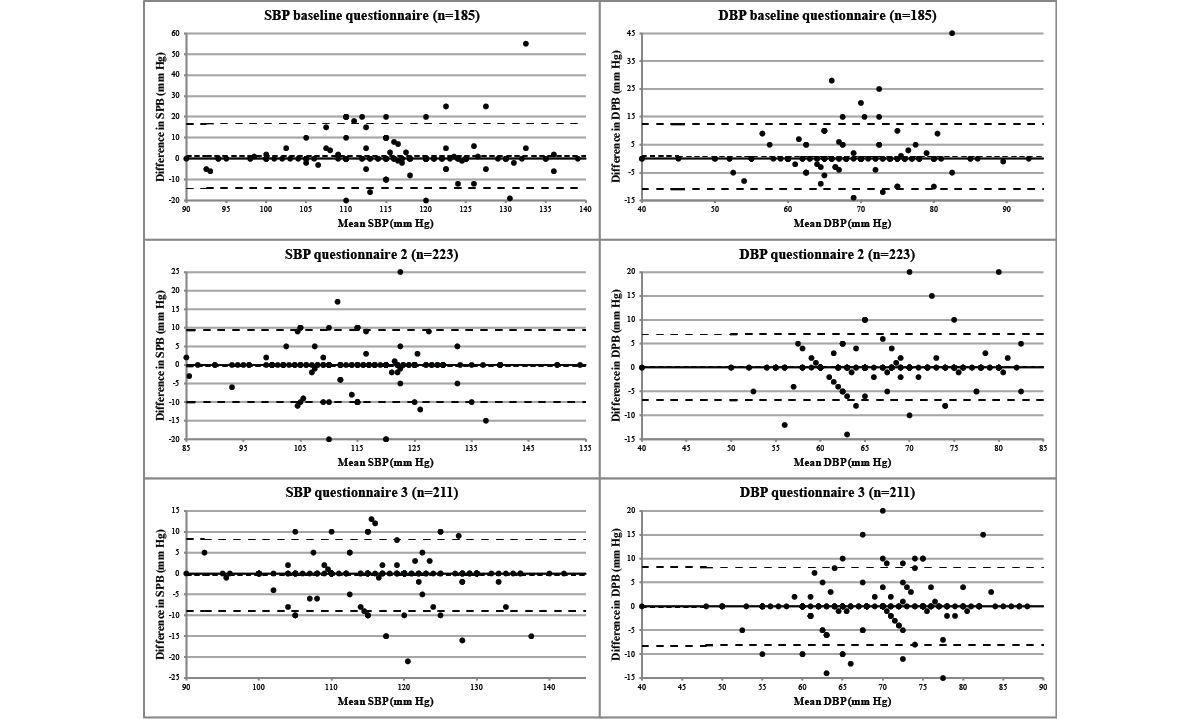
Bland-Altman plots showing the differences in reported systolic blood pressure (SBP) and diastolic blood pressure (DBP) between the 3 Web-based questionnaires and the obstetric record plotted against the mean of the 2 methods of data collection. Each data point shows one participant. The short dashed line shows the mean difference. The long dashed lines show the 95% limits of agreement (mean difference ±2 SD).

At baseline, the ICCs for SBP and DBP were 0.72 (95% CI 0.65-0.79) and 0.79 (95% CI 0.73-0.84), respectively. In the follow-up questionnaires, ICCs were substantially higher, ranging between 0.89 (95% CI 0.86-0.91; DBP in questionnaire 3) and 0.92 (95% CI 0.89-0.94; SBP in questionnaire 2). The Bland-Altman plots (Figure 3) also showed good agreement between the 2 methods of data collection with very small mean differences, ranging between 1.26 mm Hg (SD 7.72) for SBP in the baseline questionnaire and –0.04 (SD 4.09) for DBP in questionnaire 3. No trends in bias within the SBP and DBP ranges were observed. The secondary analyses, in which the date of the prenatal care visit was allowed to differ up to 5 days between the questionnaire and the obstetric record, yielded similar results (data not shown).

## Discussion

### Principal Findings

Web-based questionnaires are increasingly being used as a method of data collection in medical research. The results from the present study show that data on many chronic conditions and allergies can be validly collected among pregnant women using Web-based questionnaires with sensitivities comparable to or even higher than obstetric records. However, some overreporting of allergies was observed and absence of disease was more accurately reported than presence of disease. In addition, pregnant women were able to reliably recall blood pressure readings from the most recent prenatal care visit, especially in the follow-up questionnaires, but a substantial proportion of women could not remember their blood pressure readings at all.

### Strengths and Limitations

In addition to the relatively large sample size, the use of GP records as a reference standard to validate the Web-based questionnaire and obstetric records for chronic conditions and allergies is a major strength of this study. In the Netherlands, inhabitants are obligatory listed with one GP, who coordinates access to specialized care and always receives all relevant medical information about the patient [32]. Therefore, GP records should contain the most complete information, although inaccuracies in registration of diagnoses cannot be excluded. Other strengths of this validation study include the high consent rate (75.2%) to review medical records, the high retrieval rate of obstetric and GP records (98.3% and 76.4%, respectively), and the high willingness of PRIDE Study participants to complete questionnaires through the Internet despite the study’s mixed-mode design.

Women participating in the PRIDE Study represent a highly educated population, potentially limiting the generalizability of our results. However, women included in the validation study had a lower level of education compared to women who did not give consent for review of medical records. Previous studies on the association between maternal level of education and recall sensitivity of pregnancy-related events showed inconsistent results [33-36], indicating that imbalances in this baseline characteristic may or may not be a major threat to external validity.

Validity could not be determined reliably for a number of specific chronic conditions due to their low prevalence rates in our study population or in strata based on baseline characteristics. However, it was not feasible to increase the size of the study population because medical record abstraction is a labor-intensive process. Moreover, during the time frame of this study, only one secondary/tertiary care facility participated in the PRIDE Study. Women with certain medical conditions, including preexisting hypertension or diabetes and rheumatoid arthritis, are often referred to these facilities for prenatal care in the Netherlands. Reassuringly, only a small proportion of pregnant women (15%) start prenatal care in a secondary or tertiary care setting, mainly because of complications in a previous pregnancy [25].

### Comparison With Prior Work

For many chronic conditions that were included in our analyses, data on the validity of self-report are scarce due to differences in study populations between this study among pregnant women and previous studies, which often selected an older population with higher prevalences of cardiovascular diseases, diabetes, and cancer. However, the general pattern of a better agreement for chronic conditions that have clear diagnostic criteria than for conditions that are less well-defined observed previously [11,37,38] was also visible in our study. We observed high sensitivities and specificities for somatic diseases, but low levels of agreement for a number of nonsomatic diseases, including mental and behavioral disorders and irritable bowel syndrome. This was not only the case for data from the Web-based questionnaire, but also for data from the obstetric records. Possible causes for this variability include poor communication between the patient and the health care provider, limited health literacy of the patient, or self-diagnosis in the absence of a satisfactory medical explanation for the symptoms [39].

Surprisingly, sensitivity of the Web-based questionnaire was substantially higher for asthma (0.86) and migraine (0.90) compared to the obstetric record, whereas the specificities were comparable. The traditional self-reported modes of data collection have a sensitivity ranging between 0.55 and 0.95 (median 0.72) for asthma [10,13,14,19,40] and between 0.35 and 0.67 (median 0.51) for migraine [41-43], suggesting that Web-based questionnaires might be more suitable for detecting subjects with these conditions in epidemiologic studies than paper-based questionnaires, interviews, and obstetric records. However, future studies should confirm these findings, also taking into account the manner in which the questions about these conditions are posed.

With regard to allergies, the Web-based questionnaire also seemed to be more sensitive than the obstetric record, but at the expense of its specificity indicating that overreporting occurs with the use of the Web-based questionnaire and underreporting is present when using obstetric records. However, participants with allergic symptoms who manage their symptoms with over-the-counter medication may not be registered as allergic in GP records, resulting in a lower specificity (increased number of false positives). Therefore, skin-prick tests or serum-specific immunoglobulin E levels may be a more appropriate reference standard. In comparison with previous studies in different populations [20-23], allergies were somewhat more accurately reported in our Web-based questionnaire compared to the other self-reported modes of data collection.

Research interests in changes in blood pressure over time in relation to disease outcomes is growing (eg, [44,45]), but obtaining data on individual blood pressure readings may be challenging. Alonso et al [46] observed a low correlation between self-reported and directly observed information on SBP and DBP among 127 university graduates with an ICC of 0.35 (95% CI 0.09-0.55 and 95% CI 0.16-0.51, respectively). We are not aware of other studies reporting on the validity of self-reported blood pressure readings. In our longitudinal study, we observed a learning effect; the ICC for SBP and DBP was higher for the follow-up questionnaires than for the baseline questionnaire. Once women reported a blood pressure reading, they were very likely to report blood pressure readings in follow-up questionnaires as well. In addition, the proportion of women who could not remember their blood pressure readings decreased. As a future alternative to self-reports of blood pressure measurements conducted in health care settings, home blood pressure telemonitoring may be used to collect data on blood pressure changes over time. In addition, dedicated applications may be developed in which pregnant women could record their blood pressure readings directly after every prenatal care visit.

### Conclusions

We showed that Web-based questionnaires can validly collect data on many chronic disorders, including asthma, migraine, and thyroid disease, and also allergies among pregnant women with equal or better data quality compared to obstetric records. Although a substantial proportion of women could not remember their blood pressure readings, pregnant women who did recall the readings, recalled them well. This indicates that accurate data on general health characteristics may be collected using Web-based questionnaires in this population.

## Appendix

Multimedia Appendix 1 Screenshots of the Web-based questionnaire.

## Abbreviations

BMI: body mass index
CRF: case report form
DBP: diastolic blood pressure
GP: general practitioner
ICC: intraclass correlation coefficient
pneg: observed proportion of negative agreement
ppos: observed proportion of positive agreement
PRIDE: PRegnancy and Infant DEvelopment
SBP: systolic blood pressure
TOP: termination of pregnancy
