# Supplementary material for: Using Web-Based Questionnaires and Obstetric Records to Assess General Health Characteristics Among Pregnant Women: A Validation Study
Source: J Med Internet Res. 2015 Jun 16;17(6):e149. doi: 10.2196/jmir.3847 (PMC4526940; doi:10.2196/jmir.3847)
Supplement: Multimedia Appendix 1 [file jmir_v17i6e149_app1.pdf]

Heeft u een (andere) langdurige of chronische aandoening die is vastgesteld door een arts?

(Bijv. epilepsie, schildklierproblemen, suikerziekte, astma, migraine, depressie of ADHD)

- (•) Ja
- ( ) Nee

Vorige | Volgende

Huidige taal
Nederlands (Standaard)

Onderzoekshotline
06-13994556

Ingelogd als:
002003109

Groeps-ID:
Test (002)

Als u problemen ondervindt bij het gebruik van deze site kunt u contact opnemen door een e-mail te sturen naar help@mijnpridestudy.nl. Of u kunt direct een bericht voor ons achterlaten via de volgende link: Neem contact met ons op

Built on QMF Framework 6.0 By PMD International Contact: info@pmd-int.com

Welke langdurige of chronische aandoening(en) heeft u?

(U kunt meerdere antwoorden aanklikken)

- [x] Astma
- [ ] Bronchitis
- [ ] Hoge bloeddruk
- [ ] Verhoogd cholesterol
- [ ] Psoriasis
- [ ] Reuma
- [x] Chronisch vermoeidheidssyndroom (CVS)
- [ ] Andere aandoening(en), namelijk:
- [ ] Epilepsie
- [ ] Migraine
- [x] ADHD
- [ ] Depressie
- [ ] Angststoornis
- [ ] Suikerziekte (diabetes)
- [ ] Schildklierafwijking

Vorige | Volgende

Huidige taal
Nederlands (Standaard)
Onderzoekshotline
06-13994556

Ingelogd als:
002003109

Groeps-ID:
Test (002)

Als u problemen ondervindt bij het gebruik van deze site kunt u contact opnemen door een e-mail te sturen naar help@mijnpridestudy.nl. Of u kunt direct een bericht voor ons achterlaten via de volgende link: Neem contact met ons op

Built on QMF Framework 6.0 By PMD International Contact: info@pmd-int.com

Introductie
Deze zwangerschap
Voorgaande zwangerschappen
Zwangerschapscontroles
Uw eigen gezondheid
Vitamines
Leefgewoonten
Werk & vrije tijd
Afsluiting

Heeft u (ooit) een allergie of eczeem (gehad)?

- (•) Ja
- ( ) Nee

Vorige | Volgende

Uitloggen
Start-pagina
Mijn takenlijst

Huidige taal
Nederlands (Standaard)
Onderzoekshotline
06-13994556

Ingelogd als:
002003109

Groeps-ID:
Test (002)

Als u problemen ondervindt bij het gebruik van deze site kunt u contact opnemen door een e-mail te sturen naar help@mijnpridestudy.nl. Of u kunt direct een bericht voor ons achterlaten via de volgende link: Neem contact met ons op

Built on QMF Framework 6.0 By PMD International Contact: info@pmd-int.com

Waardoor werd de allergie of het eczeem veroorzaakt?

(U kunt meerdere antwoorden aanklikken)

- [x] Hooikoorts/pollen allergie
- [ ] Zonlicht-allergie
- [ ] Allergie voor (huis)dieren
- [ ] Allergie voor huisstof(mijt)
- [ ] Allergische reactie bij insectenbeten
- [x] Voedselallergie (bijv. koemelk, pinda's)
- [ ] Overgevoeligheid voor bepaalde metalen (bijv. nikkel, chroom of kobalt)
- [ ] Overgevoeligheid voor sommige cosmetica en parfums (ook in wasmiddelen)
- [ ] Allergie voor bepaalde geneesmiddelen
- [ ] Allergie voor pleisters
- [ ] Allergie of eczeem met een onbekende oorzaak
- [ ] Een andere oorzaak, namelijk:

Vorige | Volgende

Uitloggen
Start-pagina
Mijn takenlijst

Huidige taal
Nederlands (Standaard)
Onderzoekshotline
06-13994556

Ingelogd als:
002003109

Groeps-ID:
Test (002)

Als u problemen ondervindt bij het gebruik van deze site kunt u contact opnemen door een e-mail te sturen naar help@mijnpridestudy.nl. Of u kunt direct een bericht voor ons achterlaten via de volgende link: Neem contact met ons op

Built on QMF Framework 6.0 By PMD International Contact: info@pmd-int.com

Wanneer bent u voor het laatst op controle geweest voor uw zwangerschap?

(ongeveer) op 26-Feb-2015

Is bij deze controle uw bloeddruk gemeten?

- (•) Ja
- ( ) Nee
- ( ) Weet ik niet

Is bij deze controle uw Hb (hemoglobine gehalte) gemeten?

- ( ) Ja
- (•) Nee
- ( ) Weet ik niet

Vorige | Volgende

Uitloggen
Start-pagina
Mijn takenlijst

Huidige taal
Nederlands (Standaard)
Onderzoekshotline
06-13994556

Ingelogd als:
002003109

Groeps-ID:
Test (002)

Als u problemen ondervindt bij het gebruik van deze site kunt u contact opnemen door een e-mail te sturen naar help@mijnpridestudy.nl. Of u kunt direct een bericht voor ons achterlaten via de volgende link: Neem contact met ons op

Built on QMF Framework 6.0 By PMD International Contact: info@pmd-int.com

Zwangerschap & Gezondheid

Wat was toen uw bloeddruk?

(Bijvoorbeeld 120/80)

- (●) Mijn bloeddruk was: 110/75
- ( ) Weet ik niet

Vorige | Volgende

Huidige taal
Nederlands (Standaard)
Onderzoekshotline
06-13994556

Ingelogd als:
002003109

Groeps-ID:
Test (002)

Als u problemen ondervindt bij het gebruik van deze site kunt u contact opnemen door een e-mail te sturen naar help@mijnpridestudy.nl. Of u kunt direct een bericht voor ons achterlaten via de volgende link: Neem contact met ons op

Built on QMF Framework 6.0 By PMD International Contact: info@pmd-int.com
